# A case of recurrent gastric volvulus successfully treated with nasogastric tube‐assisted endoscopic reduction and percutaneous endoscopy‐assisted gastropexy

**DOI:** 10.1002/deo2.70079

**Published:** 2025-02-21

**Authors:** Ryuji Katoh, Eriko Katoh, Hironori Tatsuki, Fumiyoshi Saito, Masahiko Motegi, Kiyotaka Osawa, Hiroshi Saeki

**Affiliations:** ^1^ Department of Surgery Hidaka Hospital Gunma Japan; ^2^ Department of Gastroendoscopic Center Hidaka Hospital Gunma Japan; ^3^ Department of General Surgical Science Gunma University Graduate School of Medicine Gunma Japan

**Keywords:** endoscopy‐assisted gastropexy, gastric volvulus, gastric wall fixation, gastropexy, nasogastric tube

## Abstract

We report the case of a 97‐year‐old male with recurrent gastric volvulus who was successfully treated with nasogastric tube‐assisted endoscopic reduction followed by percutaneous endoscopy‐assisted gastropexy with gastric wall fixation. The nasogastric tube facilitates endoscopic reduction by enhancing the procedure's efficiency. In contrast, percutaneous endoscopy‐assisted gastropexy provides an effective, minimally invasive method to prevent the recurrence of gastric volvulus, even in patients with poor overall health. This case highlights the clinical utility of these combined approaches in managing gastric volvulus in elderly patients with significant comorbidities.

## INTRODUCTION

Gastric volvulus is characterized by the abnormal twisting of the stomach beyond its physiological range, which potentially leads to obstruction, ischemia, or perforation. Conservative treatment involving nasogastric tube placement and endoscopic reduction is typically performed in the absence of vascular compromise. However, recurrence was frequently observed.

Endoscopic reduction can be challenging in some cases, and gastropexy is often considered in recurrent cases. However, advanced age and poor health often complicate these interventions.

Here, we report a case of recurrent gastric volvulus that was successfully treated with nasogastric tube‐assisted endoscopic reduction and percutaneous endoscopy‐assisted gastropexy using Loop Fixture II (Funada's Gastropexy Device for Percutaneous Endoscopic Gastrostomy; CREATE MEDIC CO., LTD.), which achieved a favorable clinical outcome.

## CASE REPORT

**FIGURE 1:**
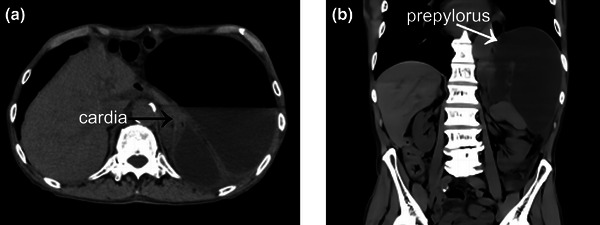
Plane abdominal computed tomography findings: (a) Horizontal section: The black arrow indicates the cardia. (b) Coronal section: The prepyloric region is inverted and displaced to the left of the aorta

The patient was a 97‐year‐old man with a medical history of hypertension and osteoporosis but no prior history of abdominal surgery. Thirty‐eight months earlier, he was admitted to our hospital for management of gastric volvulus. Owing to his advanced age, conservative treatment was administered, and the patient was followed up under observation. One month before the current presentation, the patient experienced a recurrence of gastric volvulus and underwent conservative treatment. Definitive treatment was planned following discharge; however, he presented to our hospital via emergency transport with the chief complaint of epigastric pain and abdominal distension, which had started the previous day. The patient was admitted for further evaluation and treatment. Upon admission, the patient was alert, and no significant abnormalities were observed in respiratory or circulatory dynamics. Blood tests revealed mild anemia and renal dysfunction. Levels of inflammatory markers were significantly elevated. Plain abdominal computed tomography demonstrated marked gastric distension, with the cardia and prepyloric region displaced to the left of the aorta, consistent with mesenteroaxial volvulus (Figure 1).

**FIGURE 2:**
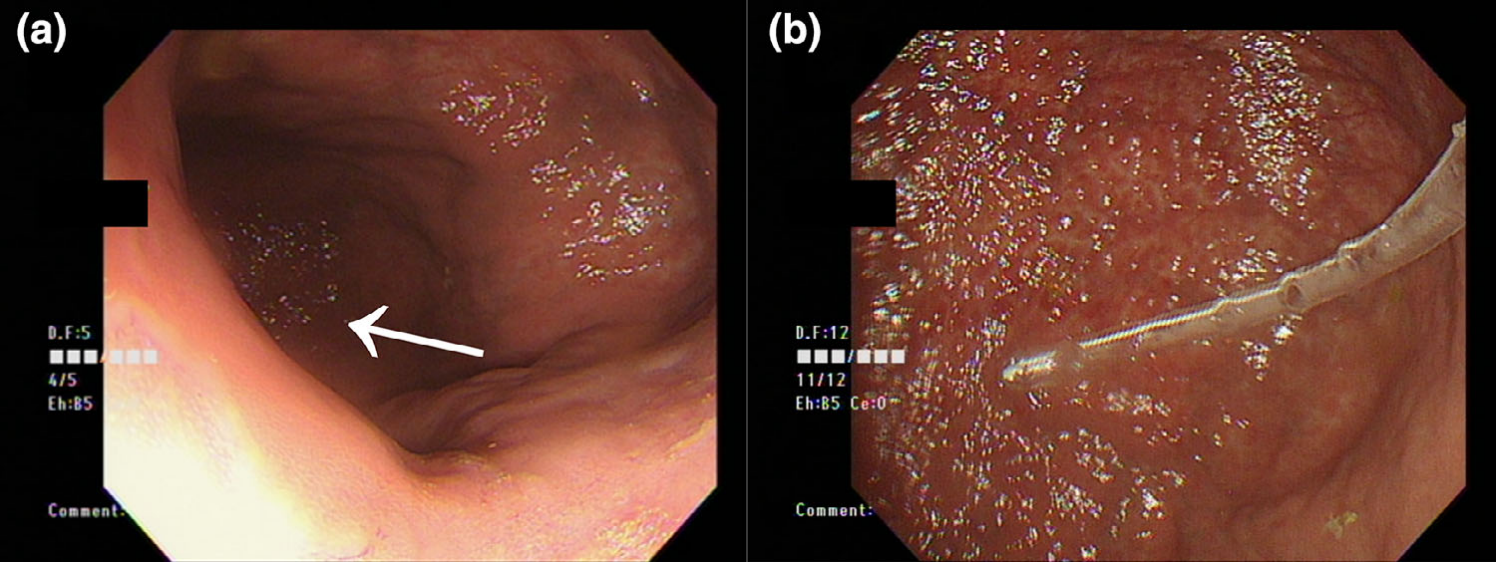
Esophagogastroduodenoscopy (during reduction): (a) Upon insertion into the stomach, the prepyloric region is observed toward the left rear of the screen (arrow). (b) After endoscopic insertion, the stomach is deflated, and a nasogastric tube is inserted. Decompression through the nasogastric tube during reduction facilitates the procedure. We performed this procedure using a CF‐H260AI (Olympus) inserted orally

An esophagogastroduodenoscopy was performed on the day of admission. Narrowing of the cardia was observed along with significant gastric distension upon insertion of the endoscope into the stomach, although the mucosal surface appeared intact. The prepyloric region was visualized on the left side. The endoscopic reduction was attempted but proved challenging because of stomach distension. A nasogastric tube was inserted, and the endoscope was advanced to the horizontal portion of the duodenum. The volvulus was successfully reduced by decompressing the stomach using a nasogastric tube (Figure 2). The patient's clinical course was favorable, and oral intake was resumed on the fifth postoperative day.

Preventive measures were deemed necessary to address recurrent gastric volvulus. However, given the patient's advanced age and preference for minimally invasive treatment, percutaneous endoscopy‐assisted gastropexy using Loop Fixture II was planned. The patient and his family were fully informed of the differences between the medical device's intended use and its application in this procedure, as well as the reported outcomes of similar treatments.

**FIGURE 3:**
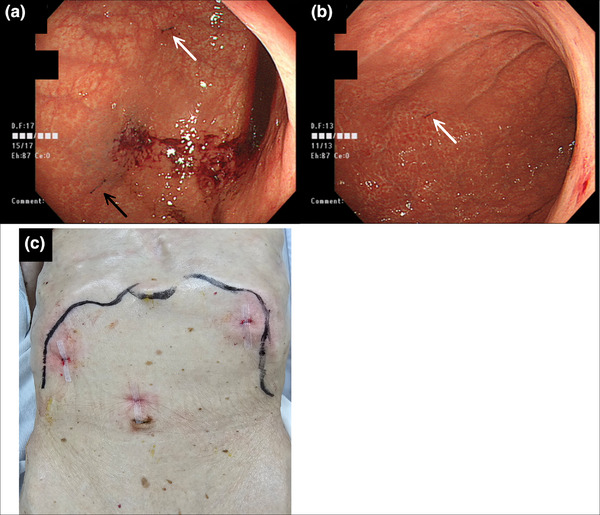
Percutaneous endoscopy‐assisted gastropexy: (a) Esophagogastroduodenoscopy: The white arrow indicates the fixation site on the anterior wall of the lower gastric body, and the black arrow indicates the fixation site on the anterior wall of the middle greater curvature. (b) Esophagogastroduodenoscopy: The white arrow indicates the fixation site on the anterior wall of the upper greater curvature. (c) External observation: The incisions were made according to the needle spacing of the Loop Fixture II, and the sites were covered with surgical tape. We performed this procedure using a GIF‐Q260J (Olympus) inserted orally

On the 17th day of hospitalization, percutaneous endoscopy‐assisted gastropexy was performed. Three fixation points were selected using transillumination for guidance^1^: the anterior wall of the lower gastric body,^2^ the anterior wall of the middle greater curvature, and^3^ the anterior wall of the upper greater curvature. Three 2‐0 nylon sutures were placed and buried subcutaneously. The incision sites were covered with surgical tape (Figure 3). The procedure, from the start of the endoscopy to its completion, took 10 min and was associated with minimal bleeding.

Postoperatively, the patient experienced persistent pain near the fixation site in the left upper abdomen (upper greater curvature). On the fifth postoperative day, the suture at this site was removed under local anesthesia by opening the wound. The pain resolved, and the patient was discharged to a care facility on the sixth postoperative day without further complications.

**FIGURE 4:**
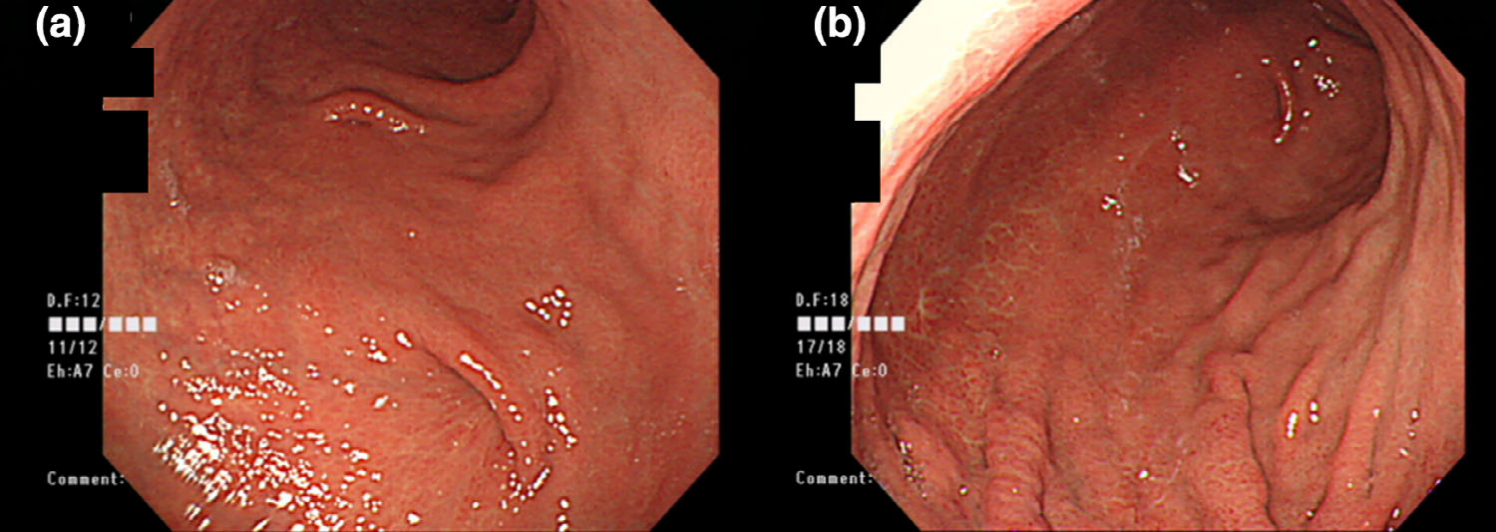
Four months post‐percutaneous endoscopy‐assisted gastropexy: (a) Traction consistent with the fixation sites on the lower and middle gastric body is observed. (b) No traction or abnormalities are noted at the upper gastric body fixation site where sutures were removed

At the four‐month follow‐up, no recurrence of gastric volvulus was observed. Esophagogastroduodenoscopy revealed traction at the fixation point (Figure 4).

## DISCUSSION

Gastric volvulus is characterized by the abnormal rotation of the stomach beyond its physiological range. An X‐ray study involving approximately 30,000 individuals revealed an incidence rate of 3.4% in children and 0.17% in adults, making it relatively rare in adults.^1^ Borchardt described the clinical triad—epigastric pain and distension, unproductive retching, and the inability to pass a nasogastric tube—as diagnostic features of gastric volvulus.^2^ Singleton classified gastric volvulus based on etiology (primary or secondary), axis of rotation (organoaxial or mesenteroaxial), direction of rotation (anterior or posterior), onset (acute or chronic), and completeness of the volvulus (complete or partial).^3^

The initial treatment typically involves decompression with a nasogastric tube and endoscopic reduction. A study of 44 patients with gastric volvulus treated conservatively over five years reported a symptom recurrence rate of 64%.^4^ This highlights the necessity of preventive measures to avoid recurrence.

Preventive strategies include surgical gastropexy and endoscopic procedures, such as percutaneous endoscopic gastrostomy.^5^ Although several reports of percutaneous endoscopy‐assisted gastropexy for gastric volvulus exist in the Japanese literature,^6^, ^7^ only two reports in English have described its use in this condition.^8^, ^9^

Two key treatment approaches were employed in this case^1^: nasogastric‐assisted endoscopic reduction and^2^ percutaneous endoscopy‐assisted gastropexy using Loop Fixture II.

The deep insertion of the endoscope into the duodenum is essential for reduction. However, distended stomachs often contain residual food, making it challenging to identify the direction of advancement. Furthermore, attempts to correct volvuli using endoscopic torque alone are often unsuccessful. Insertion of a nasogastric tube allows decompression of the stomach, which facilitates the insertion of the endoscope into the duodenum and simplifies the reduction process. As described in Borchardt's triad, nasogastric tube insertion is traditionally considered difficult in cases of gastric volvulus. However, decompression through endoscopic insertion can make the placement of the gastric tube feasible.

Loop Fixture II, which is widely used for percutaneous endoscopic gastroscopy, was used because of its simplicity and efficacy in fixing the gastric wall (Supporting Information). All three fixation points on the gastric wall were successfully secured within 10 min from endoscope insertion to completion of the procedure.

There is no consensus on the optimal number or location of fixation points or the type of suture used. Based on a review of previously reported cases along with the present case (totaling six cases), the mean number of fixation points was 3.0 (range: 2–4). In one case, the initial three fixation points were reduced to two.^6^, ^7^, ^9^ non‐absorbable 2‐0 monofilament sutures were used in all cases. The timing of suture removal varied, occurring at 14, 21, and 42 days, while in three cases, the sutures were not removed.

In an additional case, reported in an article focusing primarily on video documentation of the endoscopic procedure, specific details on the number of fixation points were not provided.^8^ However, the accompanying video demonstrates five fixation points using silk sutures at the middle gastric body.

In the present case, we selected the fixation method by considering the following two points:^1^ at least two fixation points with adequate spacing and^2^ the use of nonabsorbable monofilament sutures (nylon) to ensure long‐term stability and minimize the risk of infection or tissue reaction.

Incisions corresponding to the device needle spacing were made to ensure secure and long‐term fixation, and the sutures were buried subcutaneously. Excessive tension was avoided during the suturing to prevent tissue necrosis. In the present case, persistent pain at one of three fixation sites required suture removal under local anesthesia, which resolved the pain without subsequent recurrence. Subcutaneous burial of the sutures allows easy removal if complications arise, as demonstrated in this case. Additional fixation was performed if recurrence occurred.

During the procedure, precautions similar to those for percutaneous endoscopic gastroscopy are necessary, such as confirming the absence of interposed structures between the stomach and the abdominal wall using transillumination and the finger test. Potential complications include bleeding at the puncture site, infection, visceral injury, delayed gastric emptying due to excessive fixation, and internal herniation between the fixation points. Comprehensive preprocedural explanations to patients are crucial.

Reports on percutaneous endoscopy‐assisted gastropexy for gastric volvulus are limited, and the evidence of its long‐term efficacy is insufficient. Further case accumulation and long‐term follow‐up studies are warranted.

Percutaneous endoscopy‐assisted gastropexy offers a minimally invasive and simple approach to prevent the recurrence of gastric volvulus compared with surgical gastropexy under general anesthesia. Therefore, it is a valuable therapeutic option for the treatment of this condition.

We report a case of recurrent gastric volvulus successfully treated with nasogastric‐assisted endoscopic reduction and percutaneous endoscopy‐assisted gastropexy. The use of a nasogastric tube facilitates the reduction procedure and enhances its efficacy. Percutaneous endoscopy‐assisted gastropexy is a minimally invasive and effective method for preventing the recurrence of gastric volvulus, even in patients with poor general health.

## CONFLICT OF INTEREST STATEMENT

None.

## ETHICS STATEMENT

This case report was prepared in accordance with the ethical standards of the Declaration of Helsinki.

## PATIENT CONSENT STATEMENT

Written informed consent was obtained from the patient for the publication of this case report and accompanying images.

## CLINICAL TRIAL REGISTRATION

Not applicable.

## Supporting information



Support information.pdf
