# Supplementary material for: A case of recurrent gastric volvulus successfully treated with nasogastric tube‐assisted endoscopic reduction and percutaneous endoscopy‐assisted gastropexy
Source: DEN Open. 2025 Feb 21;5(1):e70079. doi: 10.1002/deo2.70079 (PMC11843470; doi:10.1002/deo2.70079)
Supplement: Supplementary file 1 — Support information.pdf [file DEO2-5-e70079-s001.pdf]

Loop Fixture Ⅱ

（Funada's Gastropexy Device for Percutaneous Endoscopic Gastrostomy）

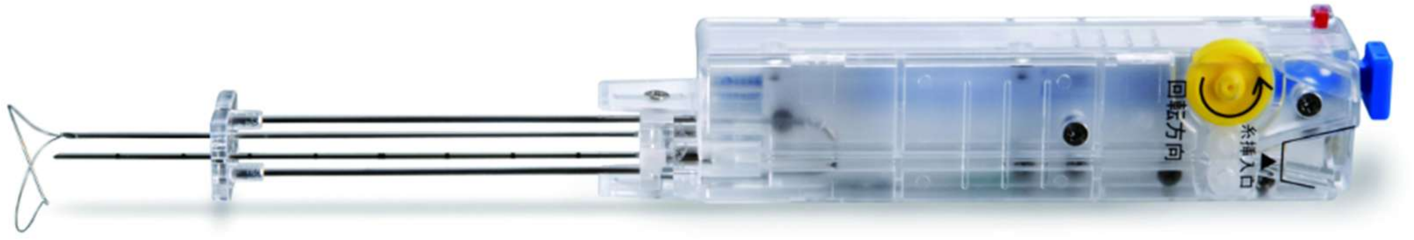

This supporting information has been created with the permission of CREATE MEDIC CO., LTD.

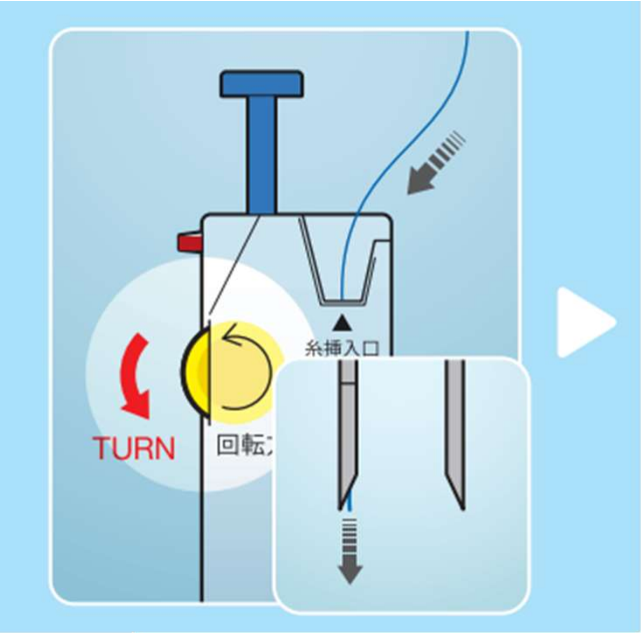

Setting the suture thread

1.Insert the suture thread in the thread insertion slot until you feel some resistance (until the suture thread comes to the roller).
2. When the suture thread comes to the thread feeding roller, rotate the thread feeding roller (yellow) downwards (towards the arrow printed on the device). You can check the feeding on the side of the device.
3. After checking that the suture thread comes out from the tip of the needle, rotate the thread feeding roller (yellow) upwards (toward the opposite direction) so that the suture thread is stored in the needle.

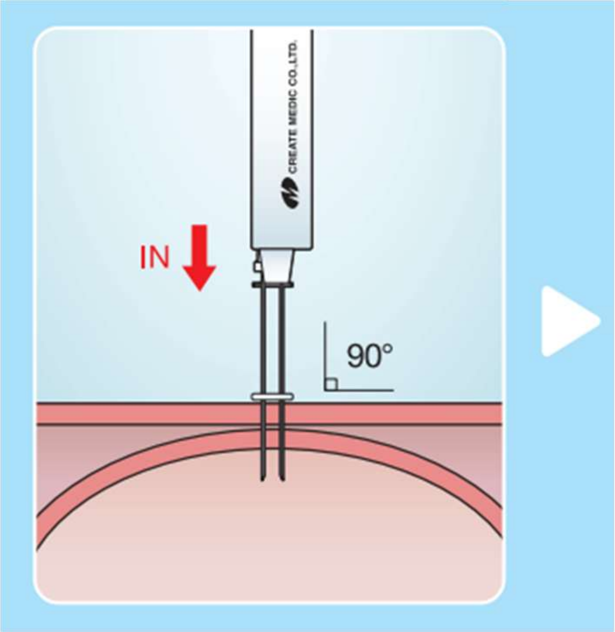

Both needles are inserted perpendicularly to the abdominal wall.

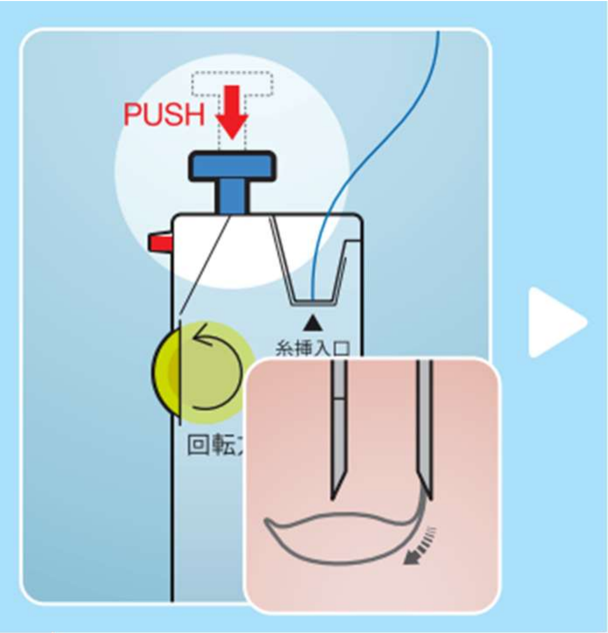

Projecting the loop

1. Push the loop insertion rod [blue] into the body of the device until it locks. (about 2cm deep)
2. Check if the loop is formed properly.

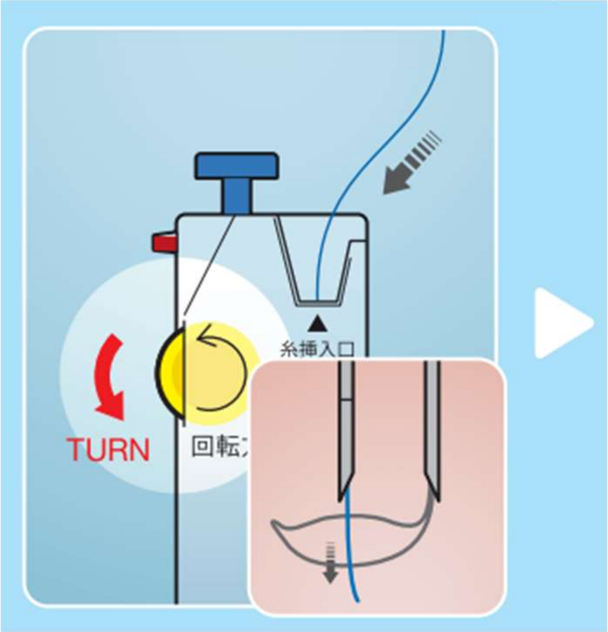

Inserting the suture thread

Rotate the thread feeding roller [yellow] downwards (towards the printed rotation direction on the device body) and pass the preset suture thread through the loop.(Pass the thread over about 2cm from the loop.)

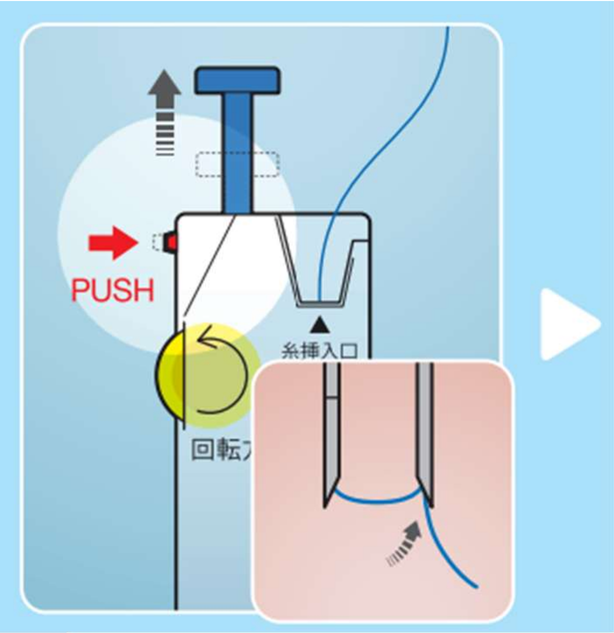

Grasping the suture thread

After checking that the suture thread passed through the loop, release the lock of the loop insertion rod [blue] by pressing the release button [red] slightly so that the loop can be stored in the needle and the suture thread can be grasped simultaneously.

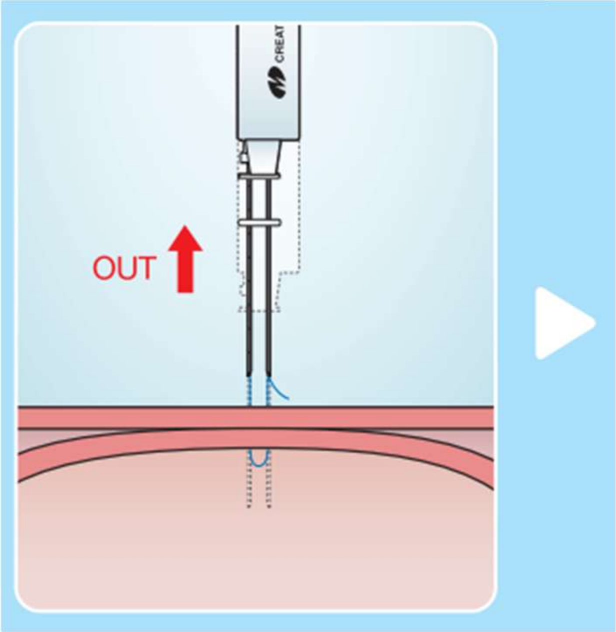

By gently withdrawing while holding the ligature thread, the ligature thread is guided out through each puncture site.

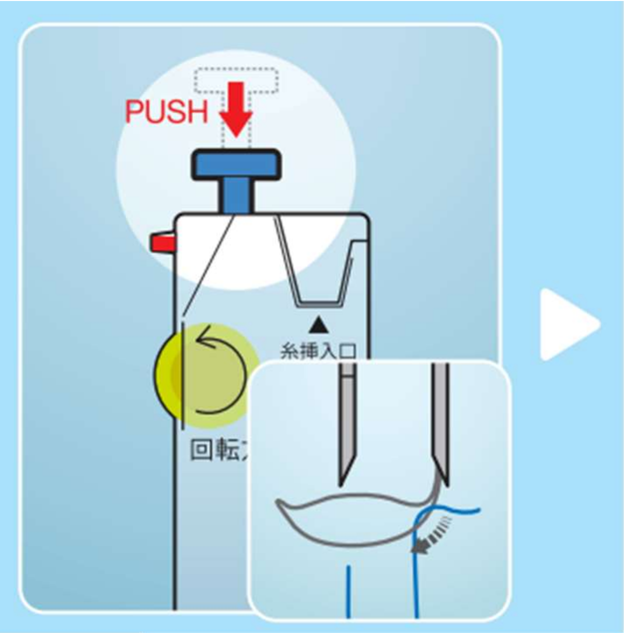

Releasing the suture thread

Push the loop insertion rod [blue] to form the loop and to release the suture thread from the loop tip fully. (Push the suture thread towards the center of the loop so that it can come off easily.)

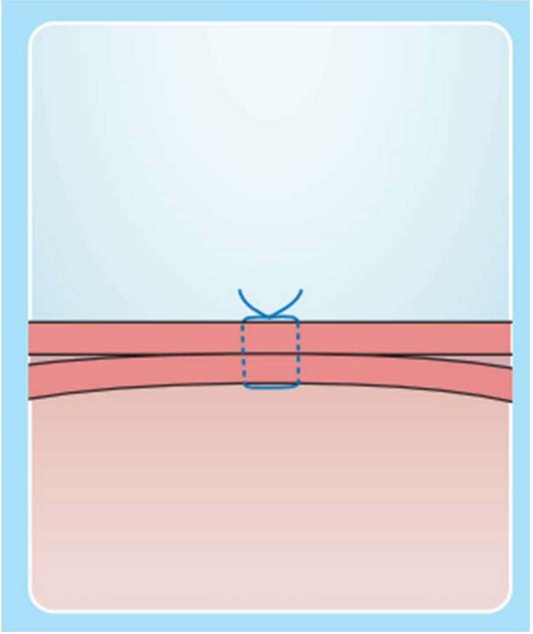

The ligature thread guided outside the body is tied, completing the fixation of the gastric wall to the abdominal wall.

For additional information, please refer to the website of CREATE MEDIC CO., LTD. linked below.

https://www.createmedic.co.jp/english/products_detail/id=421
